# Clinical and Neurobehavioral Features of Three Novel Kabuki Syndrome Patients with Mosaic *KMT2D* Mutations and a Review of Literature

**DOI:** 10.3390/ijms19010082

**Published:** 2017-12-28

**Authors:** Francesca Romana Lepri, Dario Cocciadiferro, Bartolomeo Augello, Paolo Alfieri, Valentina Pes, Alessandra Vancini, Cristina Caciolo, Gabriella Maria Squeo, Natascia Malerba, Iolanda Adipietro, Antonio Novelli, Stefano Sotgiu, Renzo Gherardi, Maria Cristina Digilio, Bruno Dallapiccola, Giuseppe Merla

**Affiliations:** 1 Laboratory of Medical Genetics, Bambino Gesù Children’s Hospital, IRCCS, 00165 Rome, Italy; francescaromana.lepri@opbg.net (F.R.L.); antonio.novelli@opbg.net (A.N.); 2 Division of Medical Genetics, IRCSS Casa Sollievo della Sofferenza Hospital, viale Cappuccini, 71013 San Giovanni Rotondo, Italy; d.cocciadiferro@operapadrepio.it (D.C.); b.augello@operapadrepio.it (B.A.); g.squeo@operapadrepio.it (G.M.S.); n.malerba@operapadrepio.it (N.M.); i.adipietro@operapadrepio.it (I.A.); 3 PhD Program in Experimental and Regenerative Medicine, Faculty of Medicine, University of Foggia, 71122 Foggia, Italy; 4 Department of Neurosciences, Child Neuropsychiatry Unit, Bambino Gesù Children’s Hospital, IRCCS, 00165 Rome, Italy; paolo.alfieri@opbg.net (P.A.); cristina.caciolo@opbg.net (C.C.); 5 Child Neuropsychiatry Unit, Department of Clinical and Experimental Medicine, University of Sassari, 07100 Sassari, Italy; valentina.pes@email.it (V.P.); stefanos@uniss.it (S.S.); 6 Neonatal Intensive Care Unit, Maggiore Hospital, 40133 Bologna, Italy; alessandra.vancini@ausl.bologna.it; 7 Child Neuropsychiatry Unit, UO Casalecchio Porretta Bologna District, 40124 Bologna, Italy; r.gherardi@ausl.bologna.it; 8 Medical Genetic Unit, Bambino Gesù Children’s, IRCCS, 00165 Rome, Italy; mcristina.digilio@opbg.net; 9 Scientific Directorate, Bambino Gesù Children’s Hospital, IRCCS, 00165 Rome, Italy; bruno.dallapiccola@opbg.net

**Keywords:** kabuki syndrome, *KMT2D/MLL2*, mosaicism, developmental delay

## Abstract

Kabuki syndrome (KS) is a rare disorder characterized by multiple congenital anomalies and variable intellectual disability caused by mutations in *KMT2D/MLL2* and *KDM6A/UTX*, two interacting chromatin modifier responsible respectively for 56–75% and 5–8% of the cases. To date, three KS patients with mosaic *KMT2D* deletions in blood lymphocytes have been described. We report on three additional subjects displaying *KMT2D* gene mosaics including one in which a single nucleotide change results in a new frameshift mutation (p.L1199HfsX7), and two with already-known nonsense mutations (p.R4484X and p.R5021X). Consistent with previously published cases, mosaic *KMT2D* mutations may result in mild KS facial dysmorphisms and clinical and neurobehavioral features, suggesting that these characteristics could represent the handles for genetic testing of individuals with slight KS-like traits.

## 1. Introduction

Kabuki syndrome (KS, MIM #147920) is a rare disorder characterized by a distinctive face, with long palpebral fissures and eversion of the lateral third of lower eyelids, short columella with broad and depressed nasal tip, prominent ears, and cleft or high-arched palate. Additional features include short stature, skeletal anomalies, congenital heart defects, renal malformations, anorectal anomalies, and abnormal dermatoglyphics [1,2].

Mutations in *KMT2D* have been identified as the main cause for KS [3], less frequent are mutations in *KDM6A* [4]. Since then, a number of studies have shown that mutations in *KMT2D* and *KMD6A* account for approximately 56–75% and 5–8% of KS cases, respectively [5].

To date, mosaic *KMT2D* mutations have been described in three KS patients [6]. Here we expand the number of mosaic KS patients by reporting three additional cases. Consistent with the published cases, mosaic *KMT2D* mutations are associated with classical KS facial dysmorphisms and clinical features, although some manifestations could be milder. KS is a multi-systemic disorder, not limited to a single organ or tissue of a common embryonic origin, and haploinsufficiency of *KMT2D* in just some cell types may be sufficient to produce deleterious phenotypic events.

## 2. Results

### 2.1. Clinical Description

**Figure 1:**
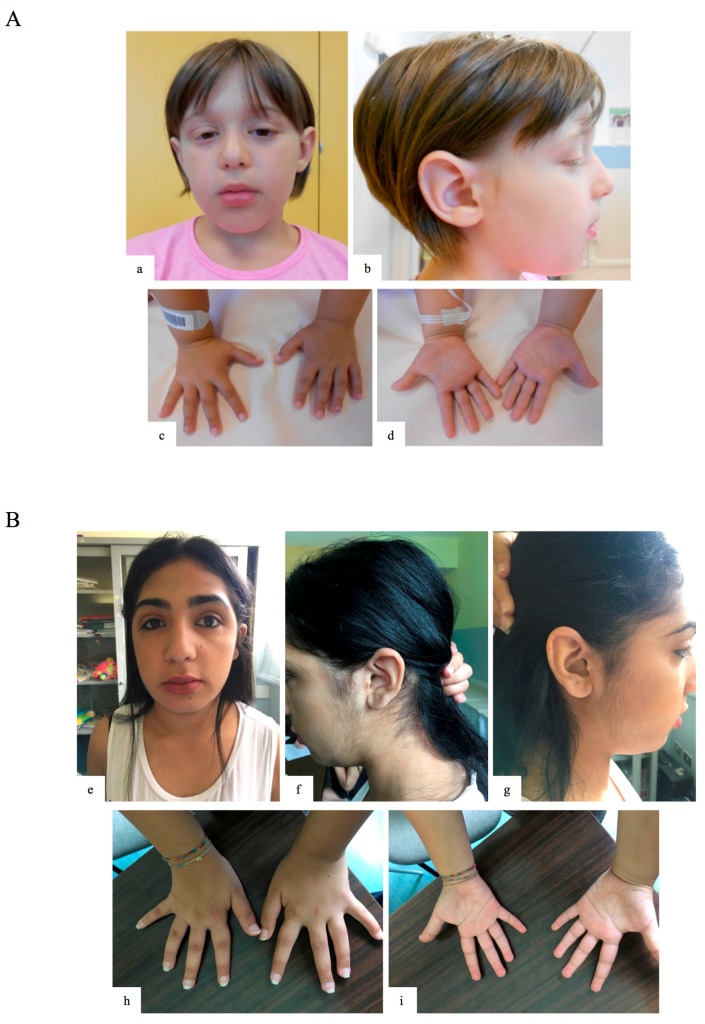
Clinical features of patients 1 and 3. (**a**,**b**) Facial features of patient 1 (GM13-3816; c.15061C=/>T, p.R5021X heterozygous mosaic *KTM2D* mutation), including sparse lateral eyebrows, long palpebral fissures with thick eyelids, ptosis, flat philtrum, thick everted lips and large dysmorphic ears with abnormal helix and large pinna; (**c**,**d**) hypoplastic proximal phalanges of 4th fingers, clinodactylous 5th fingers, and tapering fingers; (**e**–**g**) facial features of patient 3 (KB 369; c.3596_3597=/del, p.L1199HfsX7 heterozygous mosaic *KMT2D* mutation), including sparse medial eyebrows, long palpebral fissures with thick eyelids, flat philtrum, and thick everted lips; (**h**,**i**) limb/skeletal anomalies with persistent fetal pads and clinodactylous 5th fingers, and tapering fingers.

Patient 1 (GM13-3816) is the third female child of healthy non-consanguineous parents (Table 1, and Figure 1A). At birth the mother was 34 years old, the father 36. A family history disclosed learning difficulties in an older brother, depression in the paternal grandmother, a psychotic disorder in a maternal uncle, and epilepsy in a maternal niece. The baby was born by vaginal delivery at term of an uneventful pregnancy. The birth weight was 3260 g (50th centile), length 50 cm (50–75th centile), and head circumference 34 cm (50th centile). Apgar scores were 9 and 10 at 1 and 5 min. We first evaluated the patient at the age of 6.1 years for learning difficulties and facial dysmorphisms. The weight was 26 kg (97th centile), height 119 cm (75–90th centile), and head circumference 49.5 cm (10th centile). Facial anomalies included sparse lateral eyebrows, long palpebral fissures with thick eyelids, mild palpebral ptosis, flat philtrum, thick everted lips, and large prominent ears with abnormal helix and a large pinna. The proximal fourth fingers’ phalanges were hypoplastic, with clinodactylous fifth fingers. She does not have fetal fingertip pads. Tapering fingers were present. Pigmentary anomalies were also present, including partial hypochromia of eyelashes, white cutaneous spots in the trunk midline and on the dorsum, a single café-au-lait spot on the abdomen. Body asymmetry was evident, the left side of the face being larger and the left lower limb 1 cm longer in comparison to the right one. Ophthalmogic examination, audiological brainstem audiometry, electroencephalography, 2-Dimensional color-Doppler echocardiography and renal ultrasonography were unremarkable.

The neurobehavioral profile was evaluated through standardized instruments at the age of eight years and seven months. The cognitive profile was assessed using WISC IV [7]. In the verbal comprehension index (VCI) and perceptual reasoning index (PRI), the patient achieved average scores (VCI 108; PRI 87). The working memory index (WMI) and processing speed index (PSI) disclosed scores one standard deviation (SD) below the mean (WMI 82; PSI: 76). The general intellectual ability was in the average range (Total Intelligence Quozient (TIQ) 87). Adaptive behaviour was evaluated using the ABAS-II (Adaptive Behavior Assessment System—Second Edition) questionnaire parent form [8]. In the conceptual (CON) and practical domain (PR), the patient’s scores were below two SD from the mean (respectively CON: 65 and PR: 68), while in the social domain (SO) the score was one SD below the mean (SO: 81). The general adaptive composite (GAC) score was 65, two SD below the mean. The patient presented learning disabilities, with a score below one SD from the mean in the MT-2 reading task [9] in the speed and accuracy indexes, and a score two SD below the mean in the writing accuracy index of the BVSCO-2 task (Batteria per la Valutazione della Scrittura e della Competenza Ortografica—2) [10]. The psychopathological profile was investigated by psychiatric evaluation and psychodiagnostic evaluation through K-SADS-PL [11]. The patient met the criteria for generalized anxiety disorder and multiple phobias (bugs, fireworks, loud noises). She also had bizarre behaviour (talking to herself, soliloquy) and emotional dysregulation (Table 2).

Patient 2 (KB450) is the female, first child of healthy non-consanguineous parents, with the mother 33 years old and the father 37 years old (Table 1). The family history was uneventful for disabilities. The baby was born by caesarean section due to breech presentation, at term of an uncomplicated pregnancy. Birth weight was 3345 g (75th centile), length 51 cm (90th centile), and head circumference 35 cm (75th centile). Apgar scores were 9 and 10 at 1 and 5 min. A grade 3/6 L ejection systolic murmur was noted on clinical evaluation. A cardiac ultrasonography, on the first day of the patient’s life, showed a non-restrictive intraventricular subaortic defect. Kabuki syndrome’s evocative facial dysmorphisms were evident in the perinatal period, including elongated palpebral fissures extending laterally, hypertelorism, axial hypotonia and joint hypermobility, fetal finger pads, micrognathia, long and flat philtrum, low set and cup-shaped ears. Hip’s ultrasonography disclosed a right hip dysplasia. Tapering fingers were not present. Renal and brain ultrasonography, ophtalmologic examination, and audiological brainstem audiometry were all unremarkable. An X-ray at birth showed a paracardiac opacity in the right lung, 2.6 cm in diameter, interpreted by chest tomography as a small diaphragmatic hernia. The infant underwent a closure procedure of ventricular septal defects and correction of diaphragmatic hernia at one month of life with an uneventful postoperative course. Discharged at two months of life with a suspicion of KS, the patient underwent a cardiological and neuropsychiatric follow-up.

The neurobehavioral profile was assessed between six and eight years of life. The cognitive profile was evaluated using WISC IV [7]. In the WMI and PSI, the patient achieved average scores (respectively 91 and 87). In PSI and VCI, the disclosed scores were one SD below the mean (respectively 74 and 76). The general intellectual ability was one SD below the mean (Total IQ) TIQ of 84. Adaptive behaviour was evaluated using ABAS-II. In the conceptual and social domains, the patient’s scores were average (CON: 112, SO: 108), while in the practical domain the score was one SD above the mean (PR: 120). The patient presented learning disabilities with a score below one SD in the MT-2 in the reading/comprehension task [9] and below two SD in the writing accuracy index of the BVSCO-2 task [10]. Computation abilities were evaluated with the AC-MT 6–11 tests [13], reaching a total score between the fifth and tenth centile. The psychopathological profile was investigated through psychiatric evaluation, which showed mild emotional dysregulation. 

Patient 3 (KB 369) is the first female child of non-consanguineous parents (Table 1 and Figure 1B). At birth, the HCV-positive mother was 36 years old and the father 27. A younger brother has a mild intellectual disability. The baby was born by vaginal delivery at term of an uneventful pregnancy. The birth weight was 3230 g (50th centile), length 50 cm (50–75th centile). Apgar scores was 9 at 1 min. At three and a half years she was diagnosed having premature puberty and was treated with Enantone up to 12 years of age. We first evaluated the patient at five years of age, because of her tendency to isolation and poor social participation. The neurobehavioral profile was assessed at 11 years (Table 2). The cognitive profile was evaluated using WISC III [7], showing an IQ below two SD of the mean (TIQ 46, verbal IQ 58, performance IQ 46). Adaptive behaviour was assessed using ABAS-II. In the practical domain, the patient’s scores were two SD below the mean (PR: 58), while in conceptual domain the score was one SD below the mean (CON: 72). In the social domain, the score was average (SO: 88). The psychopathological profile was investigated through both psychiatric and psychodiagnostic evaluation through K-SADS-PL [11]. The patient met the criteria for generalized anxiety disorder and multiple phobias (stuffed animals, dolls and some real animals). She also had bizarre behaviour (soliloquy). At age of 12, ADOS (Autism Diagnostic Observation Schedule) Module 3 disclosed autistic-like behaviour. The weak academic competence level (reading, writing, comprehension skills) achieved by the patient did not permit a structured assessment.

At the age of 13, the family pediatrician required new medical advice for the presence of dysmorphisms. Her weight was 51 kg (>50th centile), height 153 cm (<25 centile), and head circumference 53.5 cm (<50th centile). Facial anomalies included long palpebral fissures with thick eyelids, flat philtrum, and thick everted lips (Figure 1B). Limb/skeletal anomalies were also present, including persistent fetal pads, clinodactylous of 5th fingers, and tapering fingers. Early breast development was present. Neurologic brain MRI, ophthalmologic, brainstem audiometry, electroencephalography, colour Doppler echocardiography, and renal ultrasonography evaluations were all unremarkable.

### 2.2. Mutational Analysis

**Figure 2:**
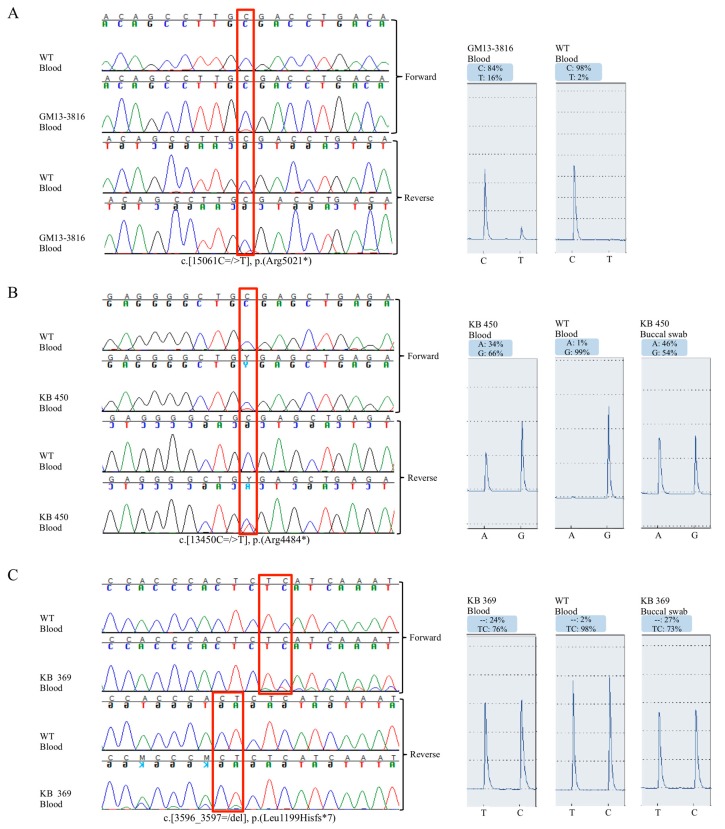
Pyrosequencing analysis confirmed the mosaic mutations. (**A**–**C**) On the left, blood DNA Sanger sequencing electropherogram of indicated patients and healthy controls. Red frame indicates the mosaic nucleotide variants. On the right, pyrosequencing peak profile of patients and control with percentage quantification of mosaic nucleotide variants. Note that for KB 450 the reverse sequence is reported for Pyrosequencing.

Patient GM13-3816 disclosed a c.15061C=/>T, p.R5021X heterozygous variant in *KTM2D* gene [14], in a small fraction of alleles from the blood cells. Pyrosequencing analysis estimated a 16% of mosaic in peripheral blood, corresponding to a ~32% mosaic in leukocytes (Figure 2A). By testing other tissue, we found 15% mutated alleles in urine cells, 2% in buccal swab, and no mosaic in hairs (data not shown).

In patient KB450 and KB369 we identified the nonsense c.13450C=/>T (p.R4484X) mutation [15], and the novel frameshift c.3596_3597=/del (p.L1199HfsX7) mutation, respectively, both in mosaic.

Pyrosequencing was used to confirm and quantify the mosaicism in different tissues. *KMT2D* c.13450C=/>T (p.R4484X) mutation was found in ~34% of peripheral blood alleles (in ~68% of leucocytes) and in ~46% of saliva cells (~92% in epithelial cells) (Figure 2B). The c.3596_3597=/del (p.L1199HfsX7) mutation was found in 20% of peripheral blood alleles (~40% in leucocytes), and in ~27% of saliva cells (~54% in epithelial cells) (Figure 2C).

Due to the unavailability of DNA we were not able to sequence any relatives from patients GM13-3816 and KB450, while both KB369 parents were negative for c.3596_3597=/del (p.L1199HfsX7).

## 3. Discussion

To date, only three KS patients with mosaic *KMT2D* deletions have been reported, two with intragenic deletions and one with a whole gene deletion, while no case has been described with mosaic *KDM6A* point mutation [6]. Here, we describe three additional KS subjects with mosaic *KTM2D* mutations. The patients with mosaic mutations described by Banka disclosed no significant difference in clinical features and cognitive profiles, in respect to non-mosaic patients [6]. Consistently, two of the three mosaic cases reported here showed distinct KS clinical characteristics, including facial features, as opposed to the GM13-3816 patient, who showed the lowest percentage of mutated cells in leukocytes (Figure 1 and Table 1). Although genotype–phenotype correlations based on few known subjects are premature, it seems plausible that both the type of mutation and the proportion of mutated cells have a direct impact on clinical outcome. Notably, five of the six known patients displayed a stature in the high–normal range (>50th centile), suggesting that mosaic mutations could have a mild effect their stature.

The neurobehavioral evaluation of patient 1 showed a learning disability, impairment of adaptive skills and psychopathological findings, such as an anxiety disorder, multiple phobias, bizarre behaviour and emotional dysregulation. A similar behavioural profile was also found in patient KB369, who was the sole patient in our cohort presenting with intellectual disability and autistic-like behaviour. A recent study of the intellectual functioning in 31 children with *KTM2D* mutations, showed IQs ranging from normal to severe disability, with most of them presented with moderately impaired IQ and a mean TIQ of 57.4 [16]. Another study of nine additional patients disclosed a similar trend, with a better mean TIQ (67) [17]. In the present study, patients GM13-3816 and KB450 showed borderline intellectual functioning (TIQ 87 and 84), while the Banka et al. [6] cases and our patient KB369 presented an obvious mild to moderate developmental delay (Table 2). Interestingly, patients displayed a more severe delay resulting from the deletion of the entire *KMT2D* gene or mutations causing a premature truncation of *KMT2D* in the first-half of the protein, predicting a shorter protein lacking the majority of *KMT2D* functional domains, including WIN and FYRC/N protein–protein interaction domains and the SET catalytic domain, therefore hampering its enzymatic activity.

The clinical examination and neurobehavioral assessment of the few mosaic KS patients did not allow us to draw at present any firm genotype–phenotype correlation. Nevertheless, some distinct facial dysmorphisms overlapping those found in non-mosaic KS patients and mild developmental delays should suggest targeted genetic testing in these subjects.

## 4. Materials and Methods

Clinical investigations and genetic analyses were approved by the institutional scientific board of the institutes involved, and were conducted in accordance with the Helsinki Declaration. DNA from KB369 and KB450 samples were deposited in the Genomic and Genetic Disorders Biobank [18]. Informed consent was obtained from family members. All children underwent complete physical and dysmorphology evaluations by the referring clinical geneticists. The clinical features of patients are summarized in Table 1.

Genomic DNA was extracted using standard procedures. Mutational analysis was carried out for patient 1 by a customized TruSeq Custom Amplicon panel using MiSeq (Illumina, San Diego, CA, USA) and validated with Sanger Sequencing. Sanger Sequencing was used to screen patient 2 and 3 as reported in [12]. PCR product was pyrosequenced twice on a PyroMark Q24 using Pyromark PCR Kit according to the manufacturer’s instructions (Qiagen, Hilden, Germany).

## 5. Conclusions

KS is a multi-systemic disorder not limited to a single organ or tissue of a common embryonic origin. The present data suggesting that the haploinsufficiency of *KMT2D* is limited to some cell types may be sufficient to recapitulate the associated phenotype. It is conceivable that screening of a larger cohort will likely identify additional cases of mosaic KS, which is so far underdiagnosed. We are persuaded that the targeted next-generation sequencing panel is the more appropriate technique for analysing this condition, since a single experiment allows full sequencing of the disease genes and the detection of somatic events, often overlooked by Sanger sequencing.

## Figures and Tables

**Table 1:** Clinical features of patients with mosaic *KMT2D* mutations.

Clinical Features	*GM13-3816*	*KB450*	*KB369*	Prevalence of *KS* Features
Gender	*F*	*F*	*F*	
Age (years)	*6.1*	*8*	*17*	
Facial anomalies	*+*	*+*	*+*	*87 ^5^*
Elongated palpebral fissures	*+*	*+*	*+*	*95 ^18^*
Sparse eyebrows	*+*	*+*	*+*	*82 ^18^*
Palpebral ptosis	*+*	*−*	*+*	*52 ^18^*
Broad nasal tip	*−*	*+*	*+*	*69 ^18^*
Thin upper and full lower lip	*+*	*+*	*+*	*71 ^18^*
Large dysmorhic ears	*+*	*+*	*−*	*90 ^18^*
Short stature	*−*	*−*	*+*	*70 ^5^*
Feeding difficulties	*−*	*−*	*−*	*82 ^5^*
Cleft palate	*−*	*−*	*−*	*37 ^5^*
Cardiac defects	*−*	*+*	*−*	*46 ^5^*
Urogenital anomalies	*−*	*−*	*−*	*42 ^5^*
IQ Impairment	*Borderline*	*Borderline*	*Moderate*	*99 ^5^*
Compromised adaptive functioning	*Mild*	*On average*	*Mild*	*99 ^5^*
Genotype	c.15061C=/>T, p.R5021 *	c.13450C=/>T, p.R4484 *	c.3596_3597=/delTC, p.L1199Hfs *7	
Leukocytes mosaicism (%)	*32*	*68*	*40*	

KS, Kabuki syndrome; F, female; * [12], # [5]; + present clinical trait, −; not present clinical trait.

**Table 2:** Summary assessment of neurobehavioral tests.

*Cognitive Profile*	GM13-3816, c.15061C=/>T, p.R5021X					KB450, c.13450C=/>T (p.R4484X)					KB369, c.3596_3597=/Del (p.L1199HfsX7)			
	Cognitive Profile													
*WISC III/IV*	***TIQ*** 87	***VCI*** 108	***PRI*** 87	***WMI*** 82	***PSI*** 76	***TIQ*** 84	***VCI*** 76	***PRI*** 87	***WMI*** 91	***PSI*** 74	***TIQ*** * 46	***VIQ*** * 58	***PIQ*** * 46	
	**Adaptive Behavior**													
*ABAS II*	***GAC*** 65	***CON*** 65	***SO*** 81	***PR*** 68		***GAC*** 115	***CON*** 112	***SO*** 108	***PR*** 120		***GAC*** 68	***CON*** 72	***SO*** 88	***PR*** 58
	***Learning Abilities***													
*Reading skills: MT-2 task*	*Speed:* <2 s.d., *Accuracy:* <2 s.d.					*Speed:* <1 s.d., *Accuracy:* <1 s.d.					*Not measurable*			
*Writing skills: BVSCO-2 task*	*Accuracy:* <2 s.d.					*Accuracy:* <2 s.d.					*Not measurable*			
*Reading comprehension skills: MT-2 task*	*Accuracy:* <2 s.d.					*Accuracy:* <1 s.d.					*Not measurable*			
	**Psychopathological profile**													
*K-SADS and psychiatric evaluation*	√ Generalized anxiety disorder √ Multiple phobias (bugs, fireworks, loud noises) √ Bizarre behavior (soliloquy) √ Emotional dysregulation					√ Mild emotional dysregulation					√ Generalized anxiety disorder √ Multiple phobias (stuffed animals, dolls and some real animals) √ Bizarre behavior (soliloquy) √ Autistic like behavior			

TIQ: total intelligence quotient, VCI: Verbal Comprehension Index, PRI: Perceptual Reasoning Index, WMI: Working Memory Index, PSI: Processing Speed Index, GAC: general adaptive composite, CON: conceptual, SO: social, PR: practical, s.d.: standard deviation, VIQ: verbal intelligence quotient, PIQ: performance intelligence quotient. * Performed with WISC III.
